# Primary pupils, science and a model bird species: Evidence for the efficacy of extracurricular science education

**DOI:** 10.1371/journal.pone.0220635

**Published:** 2019-07-31

**Authors:** Katharina Hirschenhauser, Didone Frigerio, Victoria Leithinger, Ines Schenkenfelder, Brigitte Neuböck-Hubinger

**Affiliations:** 1 University College for Education Upper Austria, Linz, Austria; 2 Core facility Konrad Lorenz Research Station for Behaviour and Cognition, University of Vienna, Grünau im Almtal, Austria; 3 Department of Behavioural Biology, University of Vienna, Austria; Universidad de Chile, CHILE

## Abstract

Beyond subject matter learning, primary science teaching aims at encouraging positive attitudes toward, and lasting interest in, science. This study tested whether the learning and science commitment of 8- to 10-year old pupils was affected by extracurricular learning opportunities involving repeated interactions with free-living northern bald ibis (*Geronticus eremita*; an endangered bird species) and professional researchers. To examine the project’s efficacy, pupils’ learning progress was monitored by repeated measures of knowledge (i) about northern bald ibises in specific, and (ii) of bird diversity in general. In addition, the children’s attitudes toward science were monitored and their dynamic mental models of northern bald ibis’ morphology and size were assessed from drawings. A total of 55 pupils from two schools were tested for eight months (before, during and after the experience). Control groups went through regular (curricular) science lessons with similar content and time investment. The extracurricular experience produced a clear learning progress with large effect sizes, which was particularly evident on a long timescale. In addition to subject matter knowledge, the project group pupils could name more bird species and expressed their views about the importance of scientific research for society in a higher proportion than control groups. Differences between schools suggest that project participation also changed the teacher’s own interest in northern bald ibises, which affected learning in the control group taught by the same teacher. Beyond the pupils’ language and reading skills, learning progress was also visible by scoring ibis-typical features in drawings; their mental models of relative size were not changed due to project participation, however. The results are discussed in reference to inducing and maintaining pupils’ attitude and interest in a topic. The study adds empirical evidence for the potentials of advancing primary science education e. g. by fostering the collaboration of schools with scientists.

## Introduction

Science education in primary schools is meant to produce learning of, and interest in, natural phenomena and environments rather than merely learning of facts from text books. It is the school’s task to pick up the personal interests of the pupils, i.e. to establish connections between their interests and the curricular requirements [1]. Children need to be enabled to observe and to learn about their environment through enquiry in order to foster a responsible relationship with nature and the environment [2]. In elementary and primary science education this may be a way to develop a positive attitude to science [3] with the aim of encouraging students to ask questions. This attitude is regarded as the overall evaluation of a topic, such as liking or disliking it [3] and/or assessing a topic as being worthwhile. Recently, Nadelson et al. [4] pointed out that the learners’ attitudes have strong effects on the dynamic changes of individual concepts and particularly on remembering relevant information. For enquiry-based learning, pupils are guided by the teacher in order to find a solution which relies on active investigation (such as processes of own observations) and to subsequently evaluate the empirical evidence for this solution [5]. Enquiry-based science instruction promotes problem-solving and active thinking in learners [6] by connecting new experiences with basic concepts from other contexts [7]. Teaching strategies that actively engage students in the learning process through scientific investigations are more likely to increase conceptual understanding than standard strategies that rely on rather passive techniques [8]. However, addressing the cognitive dimension of enquiry alone appears to be ineffective when it comes to the learners’ dynamic conceptual understanding, their motivation and their attitudes toward science [5,9]. Empirical evidence for the efficacy of adding affective dimensions and professional guidance to the curricular contents of primary science education may advance our understanding of science teaching and learning.

Curricula for primary science education attempt to support pupils in developing their skills in exploring and investigating nature through direct contact with natural objects and environments. In the context of gaining experiences with wildlife or other aspects of the natural world the Austrian national curriculum clearly encourages direct contact with nature, including the observation of animal behaviour. It also recommends fostering a knowledge of species’ diversity and interactions between organisms and their environment along with anthropogenic effects. The breadth of phenomena and knowledge required to teach these multiple aspects in a meaningful combination is a challenge for teachers, as well as for teacher education. Thus, effective science education may benefit enormously from inviting professional experts into classes [10], as well as from choosing subjects of regional relevance. The cooperation with experts may be appreciated by teachers, particularly regarding the use of wildlife as a teaching tool. However, extracurricular activities are often limited by systemic or legal matters.

Furthermore, the use of natural objects such as plants and living animals in science classrooms carries the great potential to activate learning motivation, to generate positive scientific attitudes and thus, may contribute to the children’s conceptual understanding of the nature of science [11,12]. Children have a genuine interest in natural environments and living animals, as part of their biological heritage [13]. The benefits of this for science education are increasingly recognized by school representatives [14–16]. Some recent studies confirmed the efficacy of using living animals (and plants) in science education (chickens: [17]; mice: [18]).

The presented project may be regarded as a learning opportunity, which matched the curricular demands of teaching biology in primary science education by using a model bird species [19]. The project provided formal and informal science activities in the fields of conservation, ornithology and bird behaviour. These activities were scheduled on a regular basis and were jointly supervised by the teacher and the scientific partner. The children had the opportunity for experiences on three levels, all of which may be beneficial for learning: first, they experienced close encounters with living animals, which adds an affective dimension that is beneficial for developing personal interest and a positive attitude for the subject and thus, for learning [14–18]; secondly, motivation is usually high in out-of-school learning and thirdly, they had the chance for repeated interactions with professional scientists, potentially contributing to new insights about hitherto unresolved questions regarding ibis behaviour (essentially serving as citizen scientists; [20,21]). In contrast to ‘regular’ (curricular) science classes, the encounters with professional experts and the involvement in the ibis research (“extracurricular” hereafter) allowed for emotional (i. e. animal-assisted), contextual and sociocultural experiences, i. e. contributing to current research and regional relevance of the subject.

One of the special features of this project was the experience and cooperation of school children and their teachers (and schools) with professional researchers. Typically, teachers and researchers involved in such joint activities believe that project participation will result in an intended learning progress for the children. To scrutinize this basic assumption, the learning progress of 8- to 10-year old pupils was monitored in schools which participated in the science activities over a period of eight months. A (passive) control group, i.e. pupils who attended regular (curricular) science classes and did not join the project, was used to determine the efficacy of the intervention.

The hypotheses were that (i) project participation positively influenced the learning success of pupils both in the immediate and long term; and (ii) the specific subject matter knowledge gained during the intervention also affected the children’s (lasting) interest in birds and avian diversity in their environment and, at a more general level, their attitudes toward scientific research. Finally (iii), it was also tested whether the children’s drawings before and after project participation were useful to monitor concept changes (i. e. dynamic mental models *sensu* [4]) of a bird’s morphology and size. The results presented here add empirical evidence for advancing the practice of and to encourage the involvement of extracurricular experts in primary school science education.

## Materials and methodology

The research protocol received scientific and ethical approval from the Rector’s Office of the University College of Education and the permit of the legal school authorities of Upper Austria. The children, their parents and local school authorities were informed beforehand that the results of the questionnaires will be anonymized, would exclusively serve research purposes and would not be part of the pupils’ final scores in science education. Written informed consent with the participation in this research was obtained from the parents of all participants.

The children’s learning progress for ibis specific facts, their interest in birds in general and their attitude toward scientific research were recorded across a period of eight months. Based on the children’s subject matter knowledge and drawings, we repeatedly explored the effects of project participation (i. e. emotional, contextual and sociocultural experiences) on ibis knowledge and the occurrence of dynamic changes in the children’s concepts (*sensu* [4]) of bird morphology. In a quantitative approach, learning achievements of the pupils of two classes that had participated in the scientific project (project groups) were compared to those of pupils in two same stage “parallel” classes in the same schools, which had been taught in ‘regular’ science classes (control groups).

**Fig 1:**
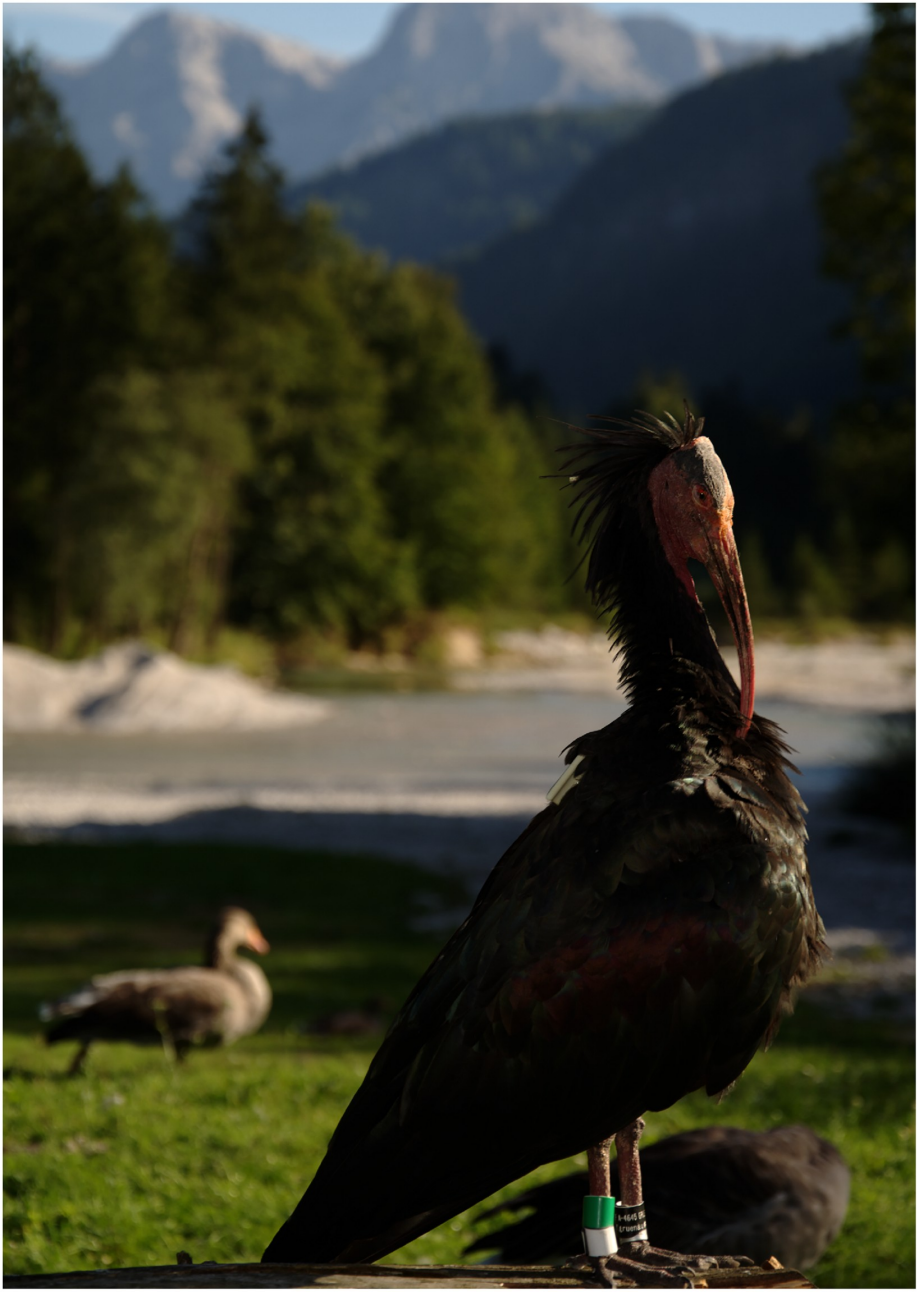
Studied species. Adult northern bald ibis (*Geronticus eremita*) marked with coloured leg rings (Picture by Verena Puehringer-Sturmayr).

At the start of the project all children were introduced to the species and were asked questions regarding ibis behaviour that would become a focus of the project. The scientists repeatedly offered insights into the monitoring of free-living northern bald ibises (*Geronticus eremita*, referred to as “ibis” hereafter, Fig 1), an endangered bird species. The occurrence of this species is a regionally unique feature in the Alm valley of Upper Austria and is an important contributor to local tourism. In the hand-reared population of this species, each bird is individually marked with a unique combination of coloured leg rings with birds habituated to the close presence of humans. The birds’ social behaviour has been investigated by the local researchers for twenty years (e.g. [19,22,23]). The colony is provided with food and shelter at the Konrad Lorenz research station, however, they are free-roaming in the region year-around. Thus, encounters with ibis foraging around the (football) fields and sitting on the roofs of houses are common in the locality. All children involved in the study were probably familiar with the species and had heard before about the birds at home, in kindergarten, at school and/or in the media given that the schools they attended were all within 10km of the valley.

## Procedure

The project offered regular contact with the ibis researchers and outdoor encounters with the birds, as well as input by visiting professional experts. The researchers offered regular scientific input into the participating classes (at least once per month) for a total of twenty weeks. At the project start they visited the children at school and introduced them to the subject: to the bird species, its specific behaviour and the research questions of the project. These were (i) to register temporal and spatial patterns of the birds in the region, (ii) to monitor whether social relationships vary across seasons, and (iii) to identify the role of social allies within the flock of free-roaming ibis [24]. The pupils were introduced and trained to identify the species, to age them (i. e. as juveniles or adults), as well as to identify individuals by the colour combination of the birds’ leg rings [25]. Following this, the children were invited to visit the Konrad Lorenz research station and encounter the ibis outdoors. There, the children had their first close contact with the free-roaming birds, were able to hand-feed the birds, and continued their training to distinguish individual birds by the colours of their leg rings and to identify behaviours (e. g. preening, foraging, bathing etc.). Children also visited the local game park, during which time they were introduced to the demands of managing the local flock and of keeping the animals in captivity. The highlight of this lesson was that the children had the opportunity to enter the aviary together with the scientists and to experience the birds’ perspective from inside the aviary. Children were also able to hone their skills in identifying individual ibis during an excursion around the area. The pupils also joined a workshop, which was held by a researcher visiting the classroom, to learn about telemetry and how to track the birds’ movements using modern tracking technology [26]. Finally, two scientists working with ibis (one person from the Alpenzoo Innsbruck, who helps run an ibis breeding programme, and one person responsible for an international ibis reintroduction initiative waldrappteam.eu) visited the schools and presented their work. The participating pupils met the researchers a total of eight times, of which they had an outdoor programme and the chance to experience the birds very closely on five occasions.

### Sample and study design

Data were collected from 55 pupils of four classes (third and fourth grade i. e. aged from 8–10 years) from two regional primary schools (school A and school B). Pupils in the project groups participated in the extracurricular activities (*N* = 27). As a control, the same data were collected from the pupils of respective, parallel, classes from the same schools (*N* = 28). Overall, the sample contained more girls than boys (on average 10%; Table 1). The two schools were located within the same district, which corresponded with the roaming area of the local ibis population. Therefore, the pupils of both schools were assumed to have had equal experience of the species prior to the investigation. The researchers invited the schools to join the project and the teachers in this study voluntarily chose to participate. In school A, the teacher of the respective parallel class was asked thereafter to serve as control group. In school B, science education was taught by the same teacher in both the project and the control group. With the teachers’ permission, all questionnaires were completed during regular lessons.

**Table 1:** Overview of sample sizes, numbers represent the gender distribution as *N* girls/boys.

	School A		School B		Total sample size
	Project group	Control group	Project group	Control group	
**Survey 1** (pre-study)	8/5	6/7	7/7	8/7	29/26
**Survey 2** (post-study immediately)	9/5	5/5	7/5	7/7	28/22
**Survey 3** (post-study long-term in third graders)	9/5	6/6	-	-	15/11

**Fig 2:**
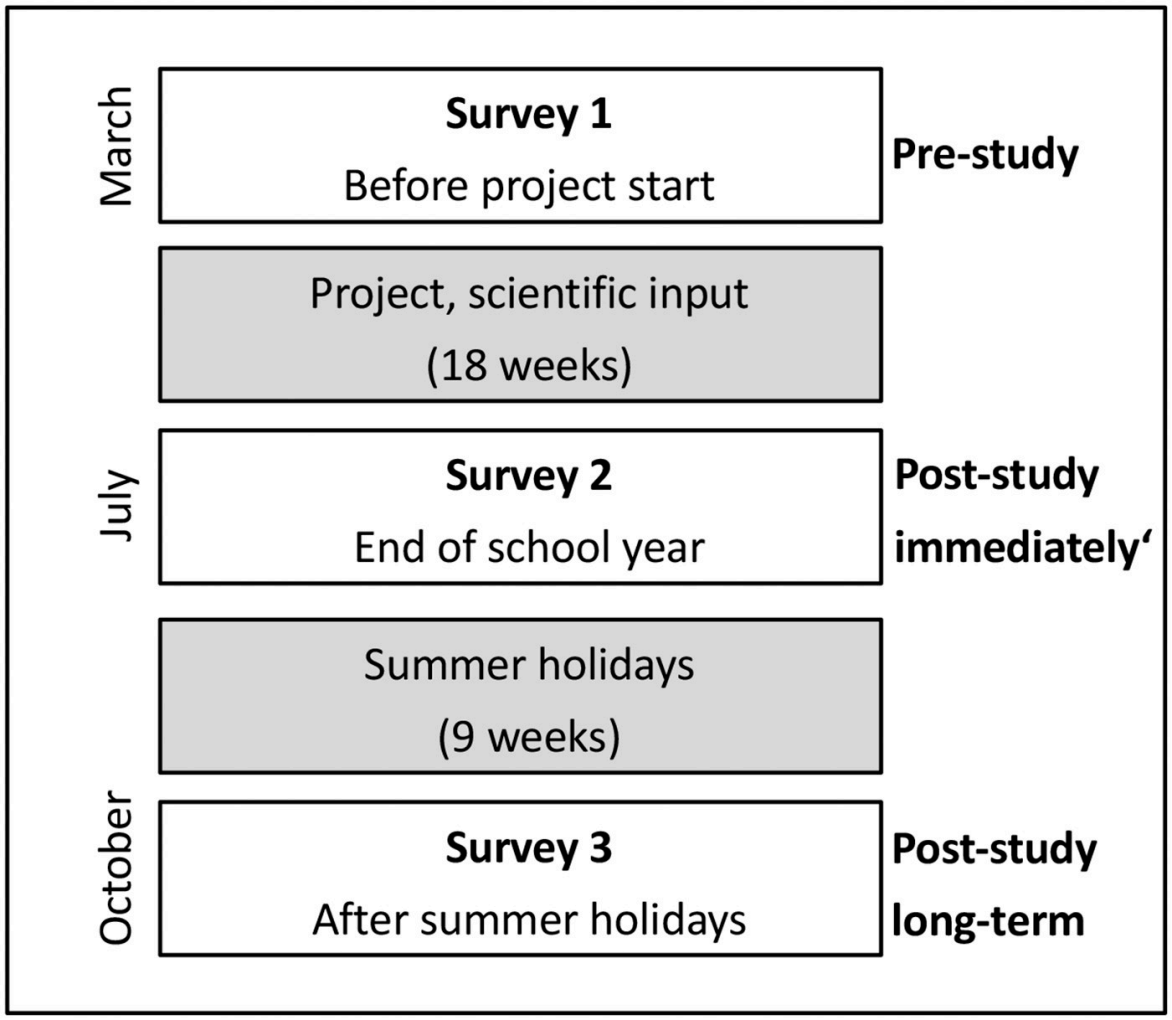
Study design. **S**chematic overview of the three repeated surveys.

Questionnaires were used to assess the children’s knowledge of the ibis, as well as their knowledge about bird diversity in general and their attitudes toward scientific research. The children completed these questionnaires before the scientific project had started (“pre-study”, March 2016, *N* = 55) and at the end of the school year (“post-study immediately”, July 2016, *N* = 50). In the subsample of third graders (*N* = 26; School A in Table 1), the questionnaires were completed for a third time in the following school year (“post-study long-term”, October 2016) to test for longer-lasting effects on the children’s knowledge (Fig 2). To note, the third survey took place after the pupils had had a period of nine weeks pause from school education during the national summer holidays. The pupils of school B were not available for the third survey because in Austria pupils change to (different) secondary schools after the fourth grade.

### Measures

The questionnaire consisted of four parts, in order to assess the children’s knowledge of subject matter (part A) and their general knowledge of bird diversity together with their attitude toward scientific research (part B; details in S1 Appendix). Furthermore, the children’s concept of ibis morphology (part C) and their mental models of relative size when drawing the birds and a football next to it (part D) were assessed from drawings. The children, in both project and control groups, were asked to complete all parts (questionnaire and drawings) three times (Fig 2).

#### Monitoring the pupils’ learning by assessing subject matter knowledge

Part A of the questionnaire may be regarded as being equivalent to an exam sheet with questions about the specific morphology, behaviour and conservation status of ibis. It contained 16 multiple choice questions together with three questions in an open format about ibis morphology, such as “Can you distinguish a male from a female ibis?”, “Does the juvenile look different than the adult bird?” or a multiple choice for the image of ibis feet as compared with images of the feet of a goose and a raven, and questions on ibis behaviour (e.g. “What is the preferred food of a northern bald ibis?”, “Do northern bald ibises live as pairs or in colonies?” and “How would you recognize the pair bond between two birds?”), as well as the species’ conservation status. The questions about the bird’s biology and behaviour were not trivial. For example, whether ibises live as pairs or in colonies depends on the season of the year, or how one would recognize the pair bond between two individuals was not immediately taught and thus, required a reflection of own experiences.

#### General knowledge about bird diversity and attitudes toward scientific research

Part B of the questionnaire aimed at assessing the children’s knowledge beyond the specific case of the ibis, i.e. their knowledge about bird diversity (“List the names of all bird species you can think of!”). At a more general level, the children’s personal perception of the value of basic research in contemporary Western societies was asked (“In your opinion, why is basic research important for society?”). For both questions the children could choose “I don’t know” in case they had no idea what to say. Both questions were in open format.

#### Pupils’ drawings of an ibis and their dynamic concepts of relative size

**Fig 3:**
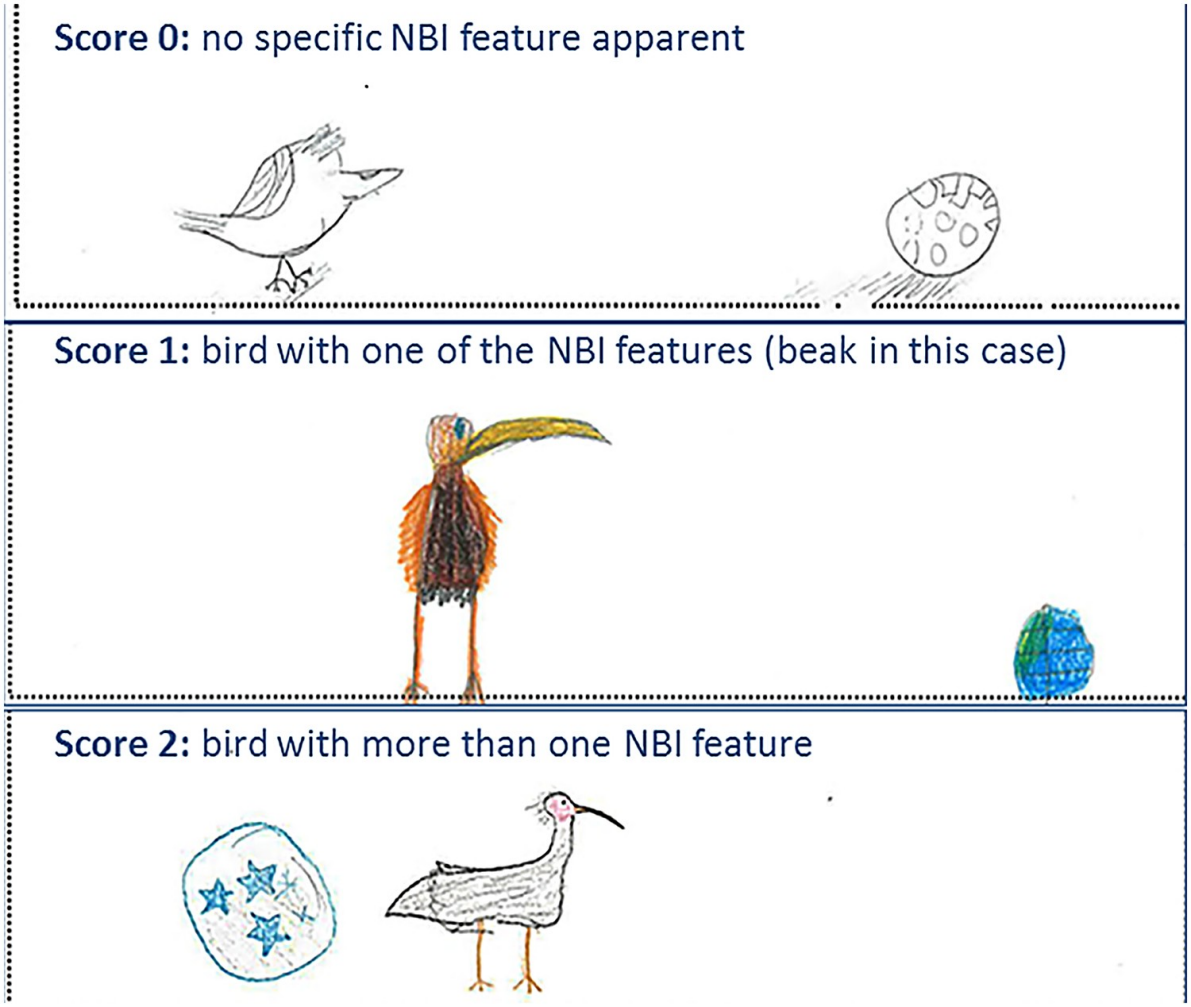
Analysing children’s drawings. Examples of the pupils’ drawings to illustrate the criteria for scoring ibis perceptibility (part C); the pupils’ concepts of the bird’s size (part D) were scored in addition on a one-zero scale, only the drawing at the bottom correctly illustrates the sizes of the bird and the football.

Accompanying each survey, the children were also asked to draw an ibis together with a football next to it. The additional drawing of a football was intended to serve as a reference for assessing the children’s dynamic concepts of relative size dimensions. From the drawings two criteria were scored by the same person (I. S.): morphological features typical for the northern bald ibis (part C) and the children’s mental models of the bird’s size (part D) as measured by the relative size difference between the bird drawing and the football (for examples see Fig 3).

To assign the scores for typical morphological features of ibis (part C) to the individual drawings, a catalogue of seven typical ibis morphological features was created. The catalogue included morphological features such as the typical shape, size and colour of the beak, feathers on the head, facial mask, bird typical toes, and leg rings (Fig 1). Scores were three-dimensional along an ordinate scale and for each drawing a score of up to two points per feature could be achieved. With the seven features a maximum score of 14 points could be assigned for each drawing.

In addition, overall perceptibility was scored in a similar way: no point was assigned when the drawing showed a bird, which was not clearly recognizable as an ibis. One point was assigned when the drawing included one of typical ibis features from the catalogue. Two points were assigned for these features when the ibis was distinctly recognizable from the drawing, i.e. more than one of the species-typical morphological features in the catalogue could be identified (Fig 3).

The pupils’ mental models of the relative size of the ibis (*sensu* [4]) as compared with drawings of a football next to the bird (part D) were scored from each drawing on a discrete one-zero scale with zero points when size relation was incorrect (e. g. when the football was clearly smaller than the bird, mid panel in Fig 3) and one point when the football and the bird were of similar size (e.g. drawing at the bottom of Fig 3).

### Statistical analysis

The assumptions of normal data distribution and equal variance were satisfied according to the Shapiro-Wilk test and the equal variance test (both with *p* ≥ 0.050). Within each of the three surveys a separate Two-Way ANOVA was employed to test for different knowledge patterns due to gender, with the factors project / control group and gender. There was no significant effect of gender on subject matter knowledge and bird diversity (Table 2), therefore data from girls and boys were combined.

**Table 2:** Between-subject effects of project participation and gender on questionnaire results (part A and part B) using Two-Way ANOVA.

	Subject matter knowledge (part A)	Bird diversity (part B)
**Survey 1** (pre-study) *N* = 55	**Project / control group**	
	***F*(1) = 9.55**	*F*(1) = 0.12
	***p* = 0.003, *d* = 0.8**	*p* = 0.267, *d* = 0.2
	Gender	
S^#	*F*(1) = 1.58	*F*(1) = 1.26
	*p* = 0.214, *d* = 0.1	*p* = 0.267, *d* = 0.1
	Interaction of the two factors	
	*F*(1,54) = 3.93	*F*(1,52) = 0.12
	*p* = 0.053, *d* = 0.4	*p* = 0.733, *d* = 0.1
**Survey 2** (post-study immediately) *N* = 50	**Project / control group**	
	***F*(1) = 24.31**	***F*(1) = 10.03**
	***p* = <0.001, *d* = 0.99**	***p* = 0.003, *d* = 0.9**
	Gender	
	*F*(1) = 1.75	*F*(1) = 0.44
	*p* = 0.193, *d* = 0.1	*p* = 0.510, *d* = 0.1
	Interaction of the two factors	
	*F*(1,49) = 0.53	*F*(1,49) = 0.12
	*p* = 0.470, *d* = 0.1	*p* = 0.727, *d* = 0.1
**Survey 3** (post-study long-term) *N* = 26	**Project / control group**	
	***F*(1) = 15.22**	***F*(1) = 7.77**
	***p* < 0.001, *d* = 0.96**	***p* = 0.011, *d* = 0.7**
	Gender	
	*F*(1) = 4.03	***F*(1) = 4.32**
	*p* = 0.057, *d* = 0.4	***p* = 0.050, *d* = 0.4**
	Interaction of the two factors	
	*F*(1,25) = 0.01	*F*(1,24) = 0.07
	*p* = 0.940, *d* = 0.1	*p* = 0.795, *d* = 0.1

Significant effects (*p*< 0.05) are depicted in bold.

**Fig 4:**
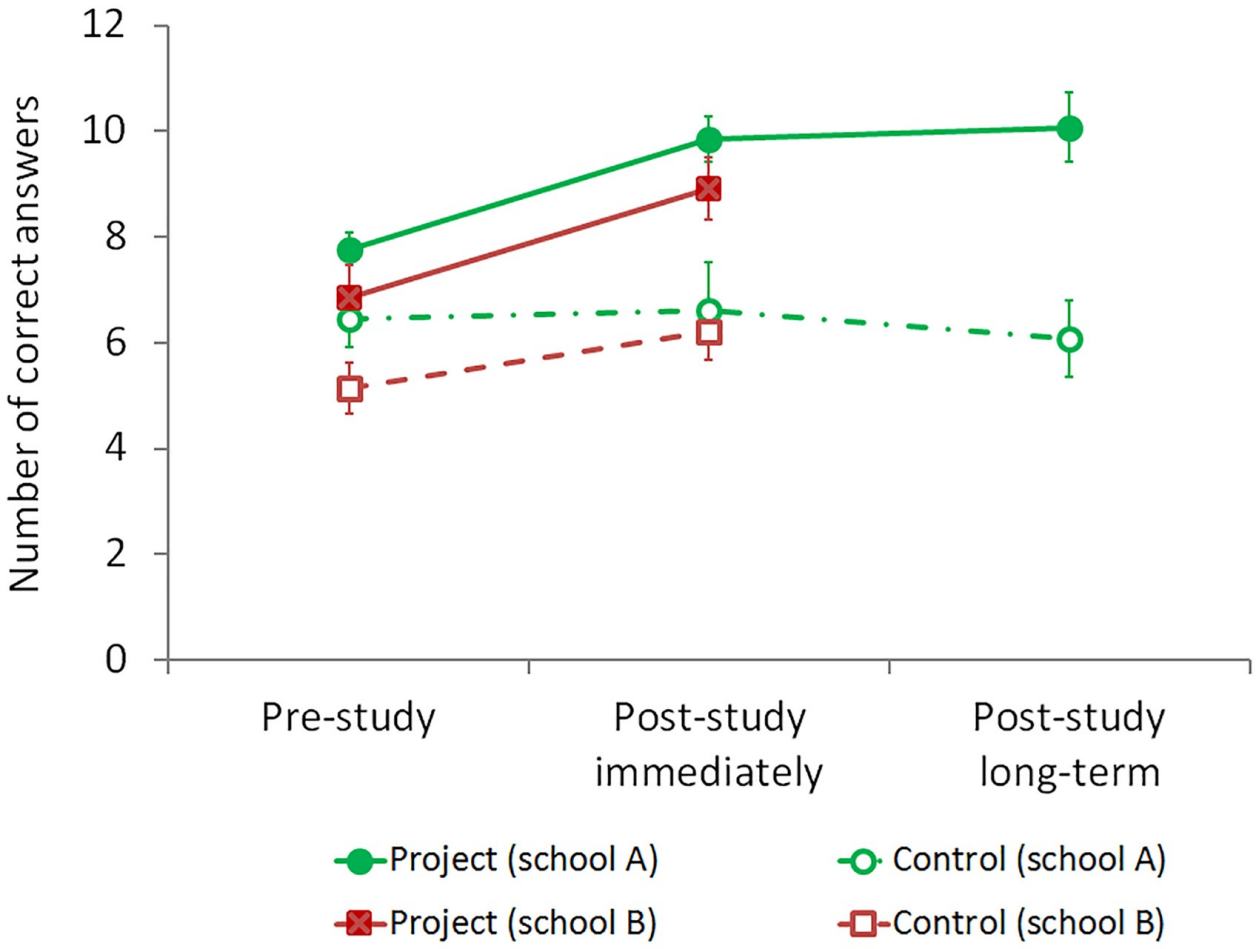
Subject matter knowledge. Patterns of recalling ibis facts in project and control groups (means ± SE).

**Fig 5:**
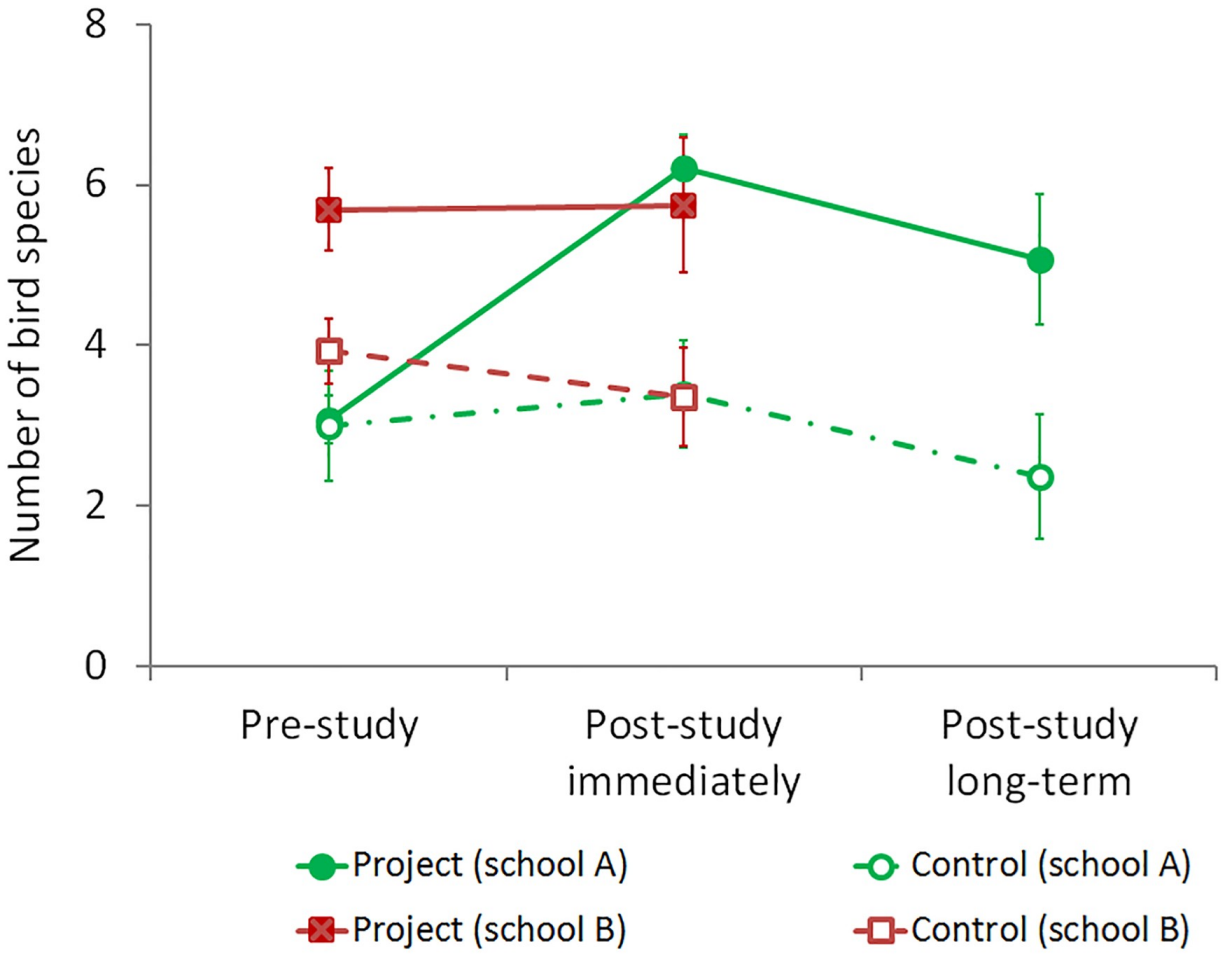
Bird diversity. Knowledge of bird diversity in project and control groups (means ± SE).

**Fig 6:**
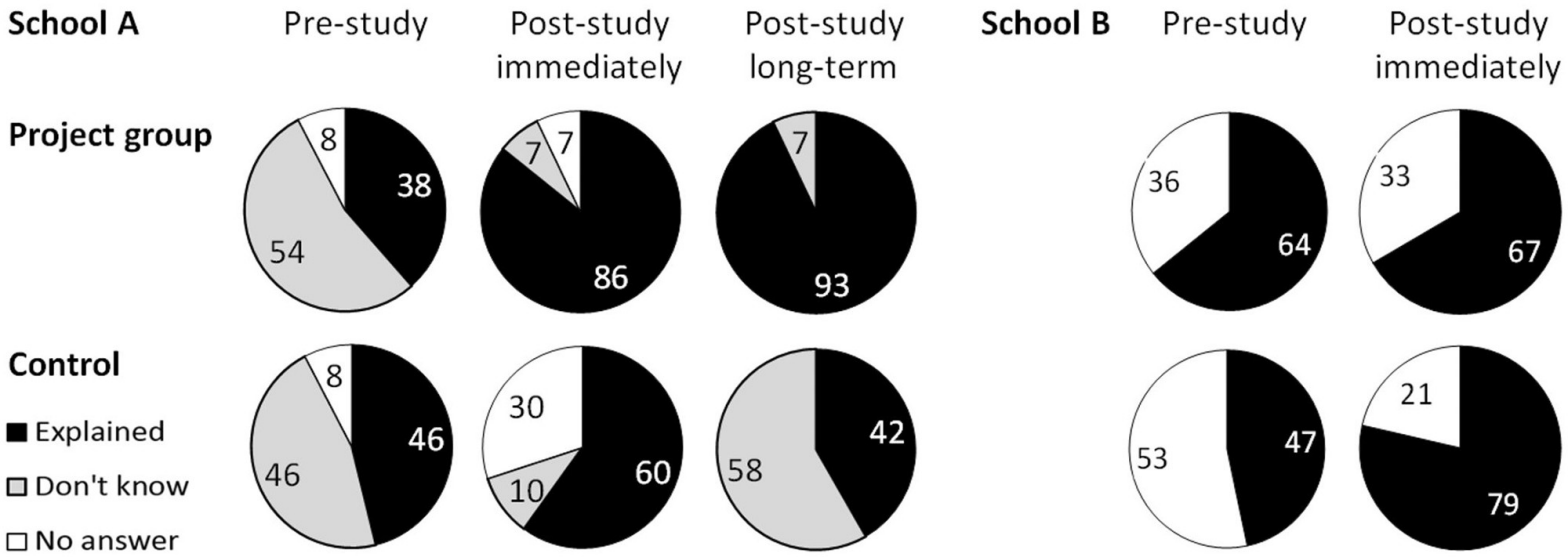
Attitudes toward scientific research. Proportions of pupils who provided an explanation of why scientific research might be important (black), of children answering with ‘I don’t know’ (grey) and of children who provided no answer to this question (open segments); school A left, school B right, project and control groups in rows, surveys in columns (before and immediately after project participation, and after the summer holidays).

**Fig 7:**
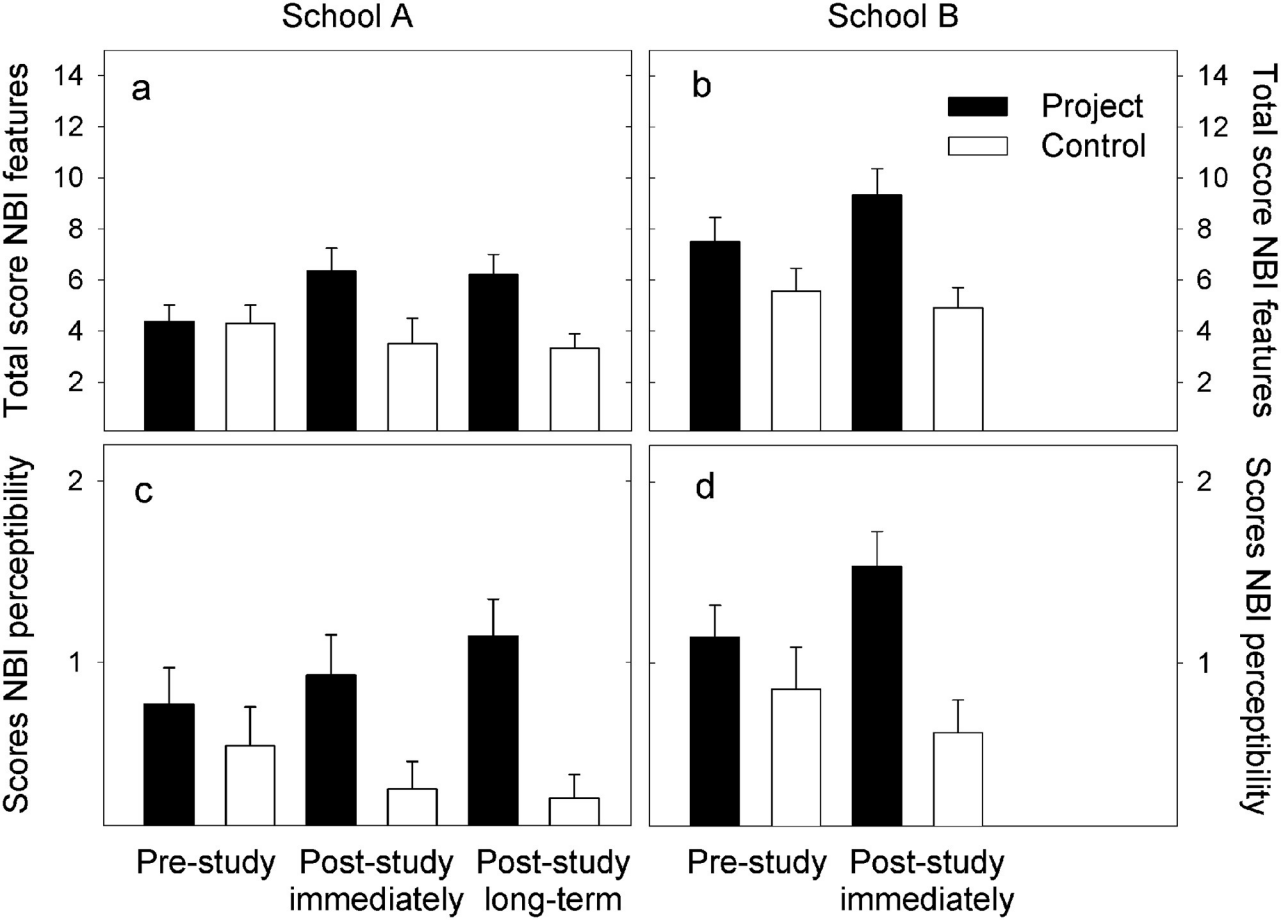
ibis typical features in pupils’ drawings. Mean (+ SE) scores for the pupils’ drawings of ibis in the three surveys in project (filled bars) and control groups (open bars). a, b (top row): ‘total scores for typical ibis features’ in the drawings; a maximum of 14 points could be achieved. c, d (bottom row): scores for the ‘overall ibis perceptibility’ in the pupils’ drawings using a discrete three-dimensional scale. (NBI: Northern bald ibis).

**Fig 8:**
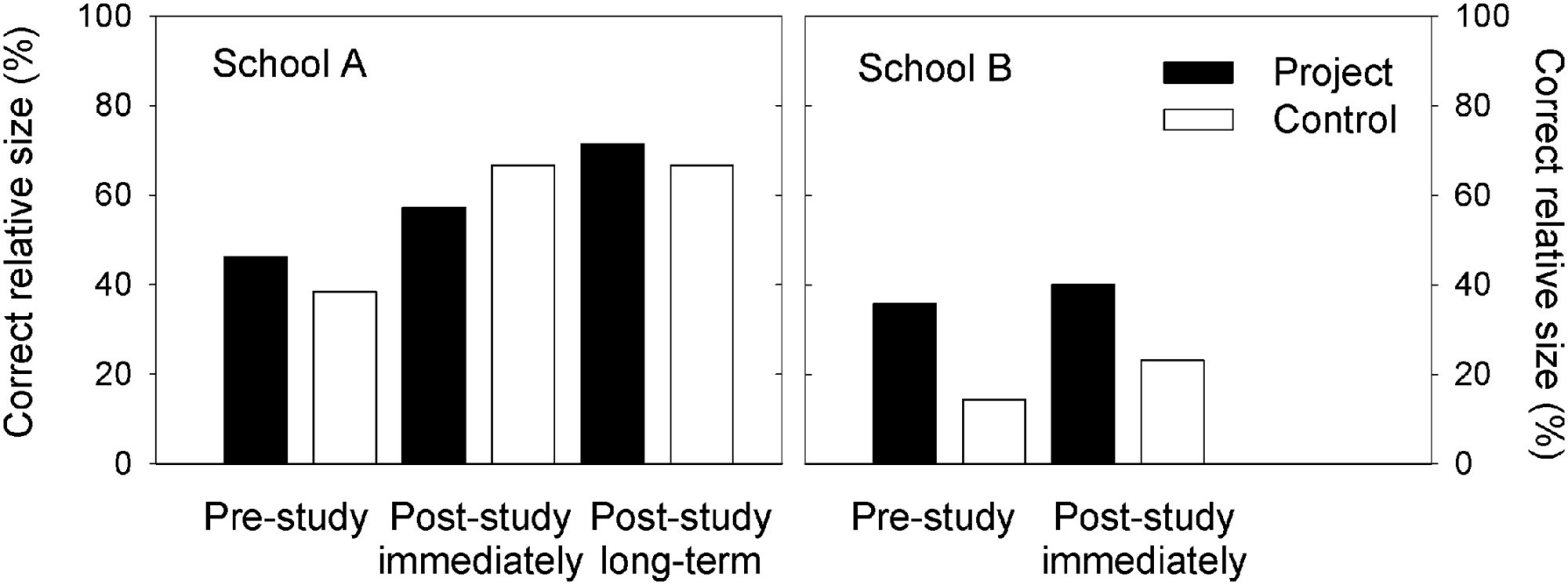
Relative size in pupils’ drawings. Proportions of drawings with correct relative size between an ibis and a football in the three surveys in project and control groups.

Plots (Figs 4–8) show the results of the two project classes and two respective control classes separately, to allow pairwise comparisons of project and control groups within schools. In addition to testing the overall difference between project and control groups, the patterns were also tested within schools (school A: df 2; school B: df 1). Results regarding changes of attitude toward science (open question format) and scores of drawings (for assessing concepts of ibis morphology and size) were analysed for each school separately.

The learning progress of children was monitored by the total number of correct answers in Part A of the questionnaire and compared between groups and within groups (with survey number as the repeated factor) using Repeated Measures-ANOVA with the factors individual and project / control group. For post-hoc pairwise comparisons the Holm-Sidak method was chosen.

Tests for dynamic concept changes as assessed from the children’s drawings were conducted in a similar way; the dependent variable was the total score for typical ibis morphological features in each of the children’s drawings (Part C) and again project / control groups and individual were set as factors. To monitor the children’s concepts of the bird’s size, the proportions of pupils drawing the bird and a football next to it with correct size relations (i.e. football is smaller than the ibis) were then compared between groups for each survey separately.

Differences were considered significant when *p* ≤ 0.050, with effect sizes *d* provided. Sigmaplot 11.0 (Jandel) was used for generating the plots and for conducting the statistical tests.

## Results

We were most interested in the effect of the intervention (project versus control group) on pupils’ learning (i. e. ibis knowledge) and attitude toward science. Therefore, we report such effects first (Table 2) followed by looking at the effect of time (pre-study versus post-study immediately and long-term; RM ANOVA) and any differences between the two schools.

### Assessing pupils’ learning by subject matter knowledge

Between groups, the subject matter knowledge was continuously higher in the project groups compared to the control groups (RM ANOVA: *F* = 13.03; df 3; *p*< 0.001; *d* = 1.0), despite the initial significant difference in knowledge between project and control groups (Table 2). Most noticeably at the longer time-scale (that is after the summer holidays), the children in the project group had more subject matter knowledge than the children in the control group (survey 3 in Fig 4).

Within groups, the children who had participated in the project activities demonstrated significantly more subject matter knowledge in the second and third surveys than in the first survey (*F* = 6.33; df 2; *p* = 0.006; *d* = 0.8). Post-hoc comparisons indicated a significant difference between the first and second surveys, while the changes from second to third survey were not significant (Fig 4).

### General knowledge of bird diversity

There was no significant difference in knowledge of bird diversity between project and control groups before the project started (Table 2). At the end of the school year (survey 2) and after the summer holidays (survey 3), children who had participated in the extracurricular project listed more names of bird species than children in control groups (Table 2) and the overall effect of project participation on bird diversity knowledge was significant (RM ANOVA: *F* = 4.93; df 3; *p* = 0.005; *d* = 0.8; Fig 5).

Nevertheless, this pattern clearly was due to progress of the children in the project group of school A (Fig 5); after the summer holidays (in survey 3, school A), all children listed fewer bird species than in survey 2; however, the children of the project group still were able to list significantly more bird species than the children of the control group in any of the three surveys (Fig 5; Table 2).

In contrast, the children in the project group of school B had high scores of bird diversity knowledge already before the project, which did not increase toward the end of the school year (on average 5.7 ± 2.7 and 5.8 ± 3.1 bird species, respectively; Fig 5). In school B, there was also no significant effect of the repeated factor (survey 1, 2 and 3; RM ANOVA: *F* = 1.58; df 2; *p* = 0.225; *d* = 0.1).

### Attitudes toward scientific research

The pupils in both schools were also asked to explain their attitude toward basic scientific research. In school A, the pupils who participated in the project had developed more differentiated attitudes toward scientific research than the pupils in the control group (Fig 6). The proportion of children in school A who provided an explanation to the question increased from 38% before the project had started (survey 1) to 86% at the end of the school year. After the summer holidays, 93% of the pupils from the project class expressed their ideas as to why scientific research might be important for society (Fig 6). The children’s explanations were diverse. For example, some were specifically addressing ibis research, i.e. ‘scientific research is good for the northern bald ibis’ or ‘to know more about the northern bald ibis’, while others provided rather general explanations, such as ‘research is important for gaining general knowledge’, or ‘to gain knowledge for further developments’. Particularly in survey 2 (i.e. at the end of the school year) some children expressed their understanding for the natural world, i.e. ‘to learn about animals and plants in their natural environment’. In the control group of school A there was some increase of the proportion of pupils providing an explanation for scientific research at the end of the school year (from 46% to 60% in survey 2); however, this increase was smaller than in the project class and in survey 3 the proportion was similar to the levels of survey 1 (42%; Fig 6).

In school B this pattern was less conclusive. Already in survey 1, a bigger proportion of the pupils who were going to participate in the project provided explanations for scientific research than in the control group. Furthermore, this level did not change markedly from survey 1 to survey 2 (Fig 6). In addition, the proportion of control group pupils who provided explanations on scientific research reached even higher levels than the parallel project class in survey 2.

### Pupils’ drawings and their mental models of relative size

There were no significant differences in the total scores for ibis drawings between project and control groups and no significant changes between surveys (RM ANOVA: *F* = 1.65; df 2,25; *p* = 0.204; *d* = 0.2). In school A, the pupils of both project and control group reached on average four of the maximum 14 points for their drawings of an ibis before the project participation had started (4.4 ± 0.6 and 4.3 ± 0.7 points, respectively). However, in survey 2 and survey 3 project group children gained higher scores for their drawings (6.4 ± 0.9 and 6.2 ± 0.8, 14% and 13% increase, respectively) than in survey 1, whereas in the control group the mean scores for drawings did not increase (-6% and -7% change from survey 1; Fig 7a).

In school B the pattern was similar; the drawings of pupils who participated in the project increased (non-significantly) between survey 1 and survey 2 (*F* = 4.12; df 1,28; *p* = 0.054; *d* = 0.4; 13% change), whereas those of the control group remained at lower scores (-4% change; Fig 7b). However, in school B the children in the project class reached high scores for their ibis drawings already before the project had started (7.5 ± 1.0). These scores were higher than those of the control group (5.3 ± 0.9; Fig 7b) and higher than those of the project class in the other school (4.4 ± 0.6; Fig 7a).

The pattern was more conclusive regarding the scores for the overall perceptibility of an ibis (Fig 7c and 7d) than based on total scores for typical ibis features (Fig 7a and 7b). With a focus on ibis perceptibility, a learning progress was recorded in both project groups; this was not observed in the control groups (Fig 7c and 7d). Initially (survey 1), the scores of drawings from pupils in the project classes were similar to those of the control groups in both schools. In survey 2 and survey 3 the scores for typical ibis features had increased in the project groups and were higher than in the control group (school A: *F* = 30.72; df 2,25; *p* < 0.001; *d* = 0.8; school B: *F* = 6.31; df 1,28; *p* = 0.019; *d* = 0.7). Thus, the changed visualisation of ibis morphology in the children’s drawings was the effect of project participation rather than due to skill development.

In contrast, the concept of relative size in the pupils’ drawings of an ibis and a football next to it was not changed due to the project intervention. The drawings from project and control groups showed similar proportions of correct size relations. This pattern was observed in both schools, however, there is no consensus between the two schools; in school A there was hardly any difference between project and control groups, whereas the children of the project group in school B were continuously better in illustrating relative size of the birds than the control group from the beginning on (Fig 8).

## Discussion

The results of this study confirmed that pupils who had participated in an animal-assisted extracurricular project showed enhanced and long-lasting learning at both the specific (i. e. in relation to ibis behaviour and morphology) and general (i. e. interest in birds and avian diversity) levels. Compared to those pupils who were taught in regular science classes, participation in the scientific project demonstrated the quantifiable potential to improve pupils’ learning and knowledge of both subject matter knowledge and a more general interest in the natural world. Furthermore, the learning and knowledge differences between project groups and regular classes were particularly evident at a longer time scale. After a break of nine weeks, children from the project groups still knew more names of bird species than the children from control groups. The results of the present study strongly complement and support the curricular demands for fostering the interest of pupils in the natural world by exploring and investigating natural objects with instructional guidance in science education.

In addition to the within-group controls, beginning with the results from survey 1 (before the project had started), we also monitored pupils in parallel classes, which did not participate in the project as between-controls. The pupils in control groups were not entirely blank of specific ibis knowledge. The birds are a local attraction, they are often seen at the meadows around the area and all children had heard about the bird species before at home and/or in kindergarten. The control groups were not merely “passive controls” (who did not experience the encounters with birds and researchers). The control “treatment” was the regular (curricular) science education, which includes lessons on birds and fauna in general, as well as on local peculiarities, such as the Konrad Lorenz research station and its scientific work. Thus, the study demonstrates patterns of learning in regular science education and compares it with the potential patterns of learning when external enhancement is allowed. Both groups spent the same amount of time in science lessons and therefore, project and control groups are comparable. The difference between groups was at the qualitative level of science instruction (i. e. context and motivation) rather than quantitative (i. e. in number of lessons).

At the functional level [27], pupils’ enhanced knowledge of bird diversity may be interpreted as an indicator of applying the knowledge of a specific subject matter to novel subjects or contexts. This demonstrates that children may be sensitized to different aspects of one bird species’ behaviour and consequently pay more attention to other avian species. The extracurricular learning opportunity may be viewed in Kline’s [28] perspective as having facilitated the transfer of abstract, generalizable knowledge and thus, providing relevant information to the children which may encourage scientific literacy (*sensu* [29], p. 34f). Also, at the affective level, the experiences when hand-feeding and interacting with the free-living birds almost certainly contributed to the pupils’ enhanced interest for their environment [12,13,15,16] and to long-lasting learning [14,17,18]. Therefore, the study adds empirical evidence that a form of collaboration between schools and research scientists, as in the present study, may be of value from a didactical perspective [10].

The efficacy of the extracurricular programme on pupils’ knowledge of bird diversity differed between the two schools. In school B the pupils of the project group—which had been selected by the teacher—knew more avian species than any of the other groups already before project participation had started (Fig 5). Moreover, the control group in school B scored highly when providing ideas for “why scientific research may be important for society” (Fig 6) without participating in the intervention (see also Discussion below). At this point it is relevant to recall that the two schools differed in an additional aspect, i. e. in school B, science education was taught by the same teacher in both the project and the control group, whereas in school A, different teachers were responsible for science education in the project and the control group. In future investigations, more attention will be paid to the random choice of project and control groups, particularly when the same teacher is involved in both, as suggested by Motz et al. [30]. Alternatively, including a third school where no intervention was carried out would add validity as a further control group. This may be regarded as a weakness of the study—however, there were large effect sizes of the overall differences between project and control groups’ learning success and knowledge about bird diversity, in spite of this (Tab. 2). Furthermore, it also points to the crucial role of the teacher as the instructor in science education. One possible explanation for the observed pattern of pupils’ attitudes toward science in the control group in school B may be an effect of the intervention on the teacher’s own attitude toward science. In agreement with the beneficial effects of the teacher’s specific knowledge of the subject matter [31]) and their pedagogical content knowledge [21,32], the teacher’s own emphasis on wildlife and their own attitude toward scientific research is likely to have contributed to the observed high proportion of pupils who provided explanations for the value of science (Fig 6) in the control group (while this was beneficial for the learning of the pupils in both groups). The impact of a teacher’s guidance on students, particularly in enquiry-based learning was also highlighted by Minner et al. [8] and Decristan et al. [5]. Potvin & Hasni [33] emphasized that the educators’ targeted efforts also affect the students’ interest, motivation and attitudes to science, although not necessarily test performance [30]. The results of the present study indicate that by fostering teachers’ attitudes toward science this may influence and improve their students’ interest and attitudes, which may subsequently foster their learning achievements.

It should be noted that a bias in the results of the control group cannot entirely be excluded in School A, as well. Initially, the researchers approached the school authorities with an invitation to participate in the project within the science education curriculum. The teachers chose voluntarily either to participate with their class or they chose not to participate. Thus, the teacher of the control group in school A did not actively choose to be involved in either the project or in data collection, on which the presented data set is based on. Considering Potvin & Hasni’s [33] argument, the lack of learning progress of the subject matter in this control group may have been due to the lack of the control group teacher’s willingness to participate. On the other hand, the pupils of the control groups in both schools had considerably lower learning success rates than the project classes (Fig 4). Therefore, we are confident that the observed effects on learning outcomes were indeed due to the extracurricular experiences of the pupils.

### Attitudes toward scientific research

The hypothesis that the extracurricular experiences influenced the pupils’ attitudes toward science was only partially confirmed. However, we argue that project participation may have changed the teachers’ attitude which consequently also affected the attitudes of pupils in the control class if they were instructed by this teacher. In school A, the project had a clear effect on the proportion of pupils providing ideas for why research might be important: the proportion of pupils’ providing their views of scientific research was continuously increasing with time in the project class and not so in the control group (Fig 6). However, in school B commitment to science increased in both project and control group.

The possibility that the collaboration with the scientists affected the teacher’s own focus points to the multidimensional nature of the presented intervention and carries potential at the level of teacher education. Disentangling the effects of the project participation on different dimensions of the teachers’ professional content knowledge [32] is one promising field that was not a focus of the presented study; recently, also Scheuch et al. [21] suggested that fostering the teachers’ expertise and scientific literacy is needed for future investigations. From the perspective of the project providing researchers, it would be of interest to learn more on how to adapt single topics to the teachers’ expectations. Servicing the teachers’ expertise and professional development would certainly contribute to a sustainable partnership between researchers and educators aiming at highly effective science education, which is feasible in practice (at least at the regional level as in the present study).

Even though not entirely comparable, both schools benefitted from the project participation. Thus, the interactions with scientists and with the free-living birds have potentials to sensitize both, the children and the teachers for various aspects of scientific research on ibis behaviour and beyond. From the educational perspective, the observed patterns indicate that the project experiences were successfully triggering the pupils’ interest and importantly, also contributing to the maintenance of the children’s interest, that is complementary to Dewey’s [34] ‘catch-and-hold theory’ of situational interest [35,36].

Also other authors have mentioned that (i) the students’ subject matter knowledge affects their attitudes toward the subject (e. g. [31]), that (ii) the teachers’ perception of and attention for science are operators for fostering scientific thinking in their students [37] and (iii) paying attention to the learners’ attitudes should be a basic concept in science education [31]. Psychological mechanisms behind the efficacy of science education also involve the learners’ self-concepts and interests. The positive relationship between self-concept, learning interest and (academic) learning outcomes has been emphasized by several authors [35,38–40]. However, most empirical studies have dealt with middle-school aged children and, with a focus on science education in the fields of physics and mathematics rather than biology. Studies on the efficacy of extracurricular science education in primary school aged children are important as they manifest (i) that a solid base for sustainable learning (and careers) and (ii) that scientific thinking potentially may be set already at this early age.

Why is it important to promote interest in science in primary school education? There is a continuing concern about the quality of science education and the lack of scientific attitudes among students; finally, personal interest has been recognized as a component of scientific literacy in the framework of PISA 2006 (Programme for International Student Assessment; [29]). Krapp & Prenzel [1] noted that students with a high cognitive potential for science did not pursue careers as scientists because they had lost their interest during school. Rather, the ones who have good self-esteem or perceive themselves as being good achievers pursue studies in science and technology (‘those who feel they can, go into the field’). However, the pupils’ personal interests for science are probably superior to their attitudes. In a meta-analysis, achievement in science was related to interest in science rather than (psychologically scaled) attitude [41]. The author suggested that positive attitude naturally *follows* successful achievement and recommended that educators should care for the attitude of the children *after* their science experiences. To resolve the problem of how to motivate children who are uninterested, science education needs to offer learning opportunities, which facilitate personal involvement, and individual (lasting) interest [36] in addition to the reaffirmation merely by test performance.

Osborne et al. [11] identified many factors that influence the pupils’ attitudes toward science and technology. Gender and the quality of teaching are most frequently investigated in the literature. In studies of interest in biology or life sciences with 6- to 10-year old children, gender is typically not (yet) a significant factor [42]. However, the base for the children’s motivation and self-concept might well be set at this age. Osborne et al. emphasized early interventions for classroom activities that may raise the pupils’ interest and motivation for studying science at school. They explicitly focussed on the affective attitudes toward science, which include the feelings, beliefs and values held about the enterprise of science, school science, the impact of science on society or scientists themselves [11]. In the presented project the animal-assisted affective component [16] of the learning subject was permitted when the children could interact with the ibis (including hand-feeding, visiting their aviary from inside and getting introduced into bio-logging) and in addition, the children had fun during these activities. Social activities, feelings of enjoyment and interest in a school subject are linked with learning success [30,43,44], which matches with the curricular desiderata of elementary and primary science education. Thus, our results support the notion that early affective experiences in science education indeed may lead to an enduring positive attitude toward science.

### Pupils’ drawings and their mental models of relative size

The hypothesis that project participation induced dynamic concept changes (*sensu* [4]) as assessed by the pupils’ drawings before and after project participation is confirmed regarding changes in the children’s mental models of a bird’s morphology, not its size. Conceptual changes of relative size (by scoring the children’s drawings of an ibis next to a football) were observed in both project and the control groups and thus, probably (i) were not due to the project participation and (ii) were not related with the children’s conception of the birds’ morphology. The lack of a conclusive pattern in drawing the correct relative size of the bird and a football rather may be subject of the pupils’ drawing abilities (see examples in Fig 3); thus, low scores may have been achieved even though they were aware of the morphological features in theory. With respect to developmental constraints, it would have been useful to have performed additional interviews with the pupils (i. e. to assess whether the drawings of 8 to 10 years old children did indeed mirror their subject matter knowledge), or to use interviews with pupils in addition to the scoring of their drawings [45]. The current results of scoring the children’s drawings should be interpreted as pilot data to explore whether this is a useful instrument to access the dynamics of mental models of (young) children. Particularly, the drawings of the children in the control groups showed that individual drawing abilities and variable talents, as well as developmental aspects may be determinants (which were not subject of this study), which affected the scores of drawings; it is likely that this was superior to visualizing learning effects of the extracurricular experiences.

On the other hand, the use of drawings to visualize children’s dynamic concept changes allows the consideration of children’s conceptual understanding independently from language and reading proficiency [5] given that some pupils are better in drawing than in verbal or written assessments. To this end, scoring illustrations is regarded as a valuable alternative and/or supplementary approach in educational research by overcoming language barriers [45–47]. However, several factors seem to modulate the drawing outcomes and need to be pinpointed in the future (e. g. the developmental patterns of drawing [45]). Moreover, conceptual development is considered a slow and gradual process [48]. The eight months of observation in the present study may not have been a sufficiently long period to visualize conceptual changes. We therefore recommend that future tests using children’s drawings for the study of conceptual developments should be extended beyond eight months.

## Conclusions

The presented study shows that regular educational practice in primary science education yet seems to be far from what scientific knowledge might offer. In order to effectively trigger attitudes, motivation and interest, children need to be engaged in activities and exposed to a variety of subject materials. Inviting scientific researchers to teach specific topics alongside the usual teachers may effectively support primary science educators. Both, pupils and teachers seemed to benefit from the interaction with scientific experts in the present study. The chance of introducing a subject from the perspectives of multiple disciplines and actors matches particularly well the demands of science education curricula and may be of interest to science educators beyond the reported Austrian case.

Further investigations, on the profound requirements for effectively conveying scientific literacy during teacher education [21,49] may contribute to better integrate extracurricular opportunities into national curricula, e. g. by redefining science education issues with regional and affective relevance. The explicit embedding of experiences with living animals inside or outside of classrooms [16] seems to be one particularly promising approach. All children have interests, motivation to explore and to engage, but not all children have had pre-school support to develop individual academic interests and motivation to learn to the best of their abilities in school. Almost twenty years ago, Hidi & Harackiewicz [36] emphasized that external interventions are important for triggering and maintaining the situational interest of unmotivated students who lack personal interest, intrinsic motivation and mastery goals for academic activities. However, empirical evidence for the efficacy of external interventions is still rare–particularly with children at primary school age.

The presented effects of extracurricular co-teaching with science experts in primary schools add to the empirical evidence for advancing the practice of science education. Moreover, the opportunity to contribute to new insights of ibis behaviour as citizen scientists was communicated to the children in project groups and probably emphasized their interest [20,50]. The identification of factors that determine whether triggered situational interest can be maintained over time is another critical issue that needs further investigation. It is challenging to disentangle the effects of single factors on learning and the development of interest and therefore, the results from quasi-experimental approaches are valuable for educational research [30]. We agree with other authors that “*empirical research is needed to inform educators about the possible multiple consequences of pedagogical variables that can be realized in practice with the available resources and time*” ([33] p. 109) and that we need experimental approaches to evaluate an action’s effect on intended outcomes [30].

## Supporting information

S1 Appendix Questionnaire on Northern bald ibis, parts A and B. (DOCX)
