# Supplementary material for: Primary pupils, science and a model bird species: Evidence for the efficacy of extracurricular science education
Source: PLoS One. 2019 Jul 31;14(7):e0220635. doi: 10.1371/journal.pone.0220635 (PMC6668844; doi:10.1371/journal.pone.0220635)
Supplement: S1 Appendix — (DOCX) [file pone.0220635.s001.docx]

**S1 Appendix**

**Questionnaire on Northern bald ibis, parts A and B**

*Part A – Subject matter knowledge about morphology, behaviour and conservation status of northern bald ibis* (* asterisks indicate open format questions)

1. Worldwide, the NBI is considered … (an endangered and rare species)
2. How can one distinguish an NBI from a greylag goose? *
3. Which image shows the feet of an NBI? (multiple choice for the images of NBI feet as compared with images of the feet of a goose and a raven)
4. Does the juvenile look different than the adult bird?
5. Can you distinguish a male from a female NBI?
6. Do northern bald ibises live as pairs or in colonies?
7. How would you recognize the pair bond between two birds?
8. Does the pair bond between a male and a female NBI hold lifelong?
9. Can the local NBI survive winter in our area?
10. In which season do NBI have their young?
11. How many young does an NBI pair usually have?
12. Where does an NBI build its nest?
13. How long does it take until the NBI chick hatches from an incubated egg?
14. Which of the images shows an NBI beak?
15. What is the preferred food of an NBI?
16. Why do NBI have such a long beak? *
17. Where do NBI sleep?
18. Do NBI make friends?
19. How would you recognize whether two birds are ‘friends’? *

*Part B - knowledge about bird diversity (both questions in open format)*

1. List the names of all bird species that come to your mind!
2. In your opinion, why is basic research important for society?
